# Systematic Reviews of Genetic Association Studies

**DOI:** 10.1371/journal.pmed.1000028

**Published:** 2009-03-03

**Authors:** Gurdeep S Sagoo, Julian Little, Julian P. T Higgins

## Abstract

Gurdeep S. Sagoo and colleagues describe key components of the methodology for undertaking systematic reviews and meta-analyses of genetic association studies.

The past decade has witnessed growing interest in genetic predisposition to common diseases, and along with rapid advancements in high-throughput genotyping technology, has resulted in a tremendous amount of published epidemiological evidence on gene-disease associations. Reported genetic associations with common diseases have become numerous and are mostly of small magnitude [1]. With this growth in evidence has come an increasing need to collate and summarize the evidence in order to identify true genetic associations among the large volume of false positives [2]. Convincing evidence of true association therefore requires careful control over potential biases and chance effects. Control over bias is important both in study design [3] and in considering the selective availability of data on associations that have been examined [4]. Because most genetic associations are small, large sample sizes are necessary for their detection, especially when many genetic variants are investigated simultaneously, as in genome-wide association studies. Furthermore, replication of findings in independent data sets is now widely regarded as a prerequisite for convincing evidence of association. Thus, multiple studies from several independent groups exist.

Systematic reviews and meta-analyses have become a common approach to summarize gene-disease associations [5]. However, as apparently inconsistent results and interpretations of syntheses of evidence on the same genetic association have been reported [6], it is useful to lay out issues in the methods and conduct of these types of research studies. The Human Genome Epidemiology Network (HuGENet) has developed “HuGE reviews” (typically systematic reviews) as the cornerstone of an online resource containing the cumulative and changing information on epidemiologic aspects of human genes [7]. Many of these contain meta-analyses, or statistical syntheses of the findings from multiple studies. In this article, we describe some key components of the methodology for undertaking systematic reviews and meta-analyses of genetic association studies. Detailed discussions of these issues and further recommendations can be found in the HuGE Review Handbook [8] (see also Box 1). Additional detailed guidance on conducting systematic reviews is available in the Cochrane Handbook for Systematic Reviews of Interventions [9] and The Handbook of Research Synthesis [10].

## Why Systematic Reviews?

**Figure 1:**
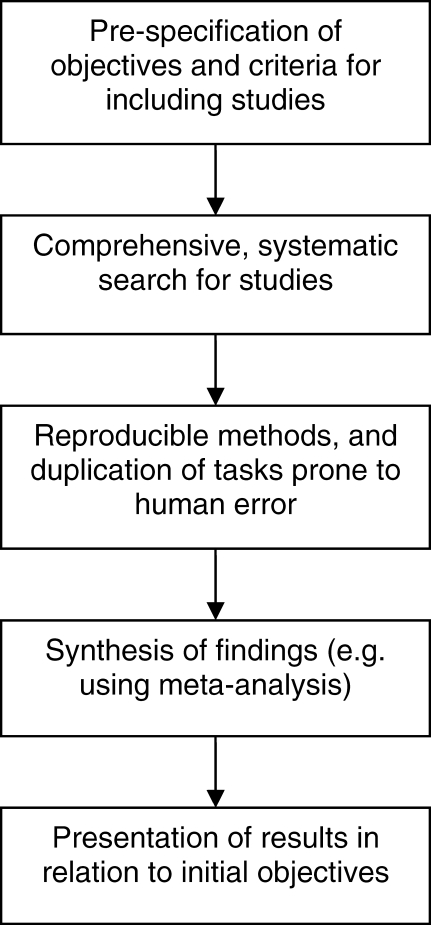
Process of a Systematic Review

Traditional (narrative) reviews are subjective, and as such have a number of disadvantages that lead them to being more prone to bias and error [11]. Narrative reviews rarely state how studies were selected for inclusion, how (or whether) their quality was assessed, or how the findings from multiple studies were synthesized in order to draw conclusions. This can lead to a review that supports and reinforces the author's view, which can be misleading. In contrast, systematic reviews are designed as rigorous research studies. They allow a more objective appraisal of the evidence than a narrative review by aiming to identify, critique, and synthesize evidence from all relevant existing studies on the topic in question using predefined methods. Figure 1 outlines the general processes involved in a systematic review.

**Summary Points**

- Systematic reviews of genetic association studies are preferable to narrative reviews, since they make the processes more transparent.
- Objectives, criteria for including studies, and methods for the systematic review should be stated in advance, ideally by writing a protocol.
- Comprehensive searches for studies should include more than one bibliographic database, as well as sources of grey literature such as online data repositories.
- Data should be extracted from reports independently by at least two people; primary study authors should be contacted for missing data or for issues that are unclear from reports.
- It is important to assess the validity of each included study, although convincing evidence of what study characteristics are most important is lacking.
- Meta-analysis of effect sizes (e.g., odds ratios) should be performed where possible and appropriate, acknowledging any heterogeneity and the potential for bias within included studies (e.g., poor validity, selective reporting) and in the data set as a whole (e.g., publication bias). Studies (rather than reports) should be the unit of analysis, to ensure data are not double-counted.

**Box 1: Development of This Guidance**

The guidance in this paper stems primarily from the Human Genome Epidemiology Network (HuGENet) HuGE Review Handbook. This document arose from a two-day discussion workshop held in Cambridge, United Kingdom, on November 2–3, 2004. In attendance were 27 participants with expertise in genetic epidemiology, clinical medicine, public health, meta-analysis, systematic review methods, searching, and statistical methods. The discussions built on previous iterations of guidance from the HuGENet Executive Committee. Speakers at the workshop were asked to comment on current methods, on empirical evidence currently available to guide methods, and on current uncertainties. A writing committee was appointed to produce the Handbook, under the editorship of Julian Little and Julian Higgins. The Handbook was influenced heavily by the Cochrane Handbook for Systematic Reviews of Interventions, which has a long history of evolution and is also based on empirical evidence where available.

The guidance has been updated for this paper to reflect developments in technology and methodology. Gurdeep Sagoo was invited to join to bring a new perspective, given his experience of undertaking HuGE systematic reviews. Passages about genome-wide association studies were added at the request of the current journal editors and are based on the consensus of the authors. The development of the guidance benefits from being the broad consensus (in 2004 to 2006) of numerous individuals with an interest in systematic reviews, but does not claim to be authoritative or to reflect the current views of all people listed below.

**Writing Group for the HuGE Review Handbook**

Molly Bray, Julian Higgins, John Ioannidis, Muin Khoury, Julian Little, Teri Manolio, Liam Smeeth, Jonathan Sterne.

**Participants at the 2004 Workshop**

Betsy Anagnostelis, Adam Butterworth, John Danesh, Carol Dezateux, Doug Easton, John Gallacher, Marta Gwinn, Julian Higgins, John Ioannidis, Muin Khoury, Sarah Lewis, Julian Little, Teri Manolio, David Melzer, Cosetta Minelli, Paul Pharoah, Georgia Salanti, Simon Sanderson, Liam Smeeth, Lesley Smith, Jonathan Sterne, Donna Stroup, Emanuela Taioli, John Thompson, Simon Thompson, Neil Walker, Ron Zimmern.

Data for systematic reviews are typically collated primarily from publications, often supplemented by data from correspondence with authors of the original studies. In the field of human genome epidemiology, however, the ability to look at increasing numbers of markers prevents the publication of summary data for each genetic variant in a traditional journal format. A variety of sources may therefore be used, including the published literature, online supplementary materials, online databases, and study authors. An increasingly popular technique is the meta-analysis of individual participant data through the collaboration of a consortium of investigators. This is likely to minimize bias compared with a literature-based systematic review, but possibly at the expense of precision, since a comprehensive systematic review may include evidence from studies that are not involved in the consortium. The two approaches are complementary to one another, and some of their potential advantages and disadvantages are summarized in Table 1.

**Table 1:**
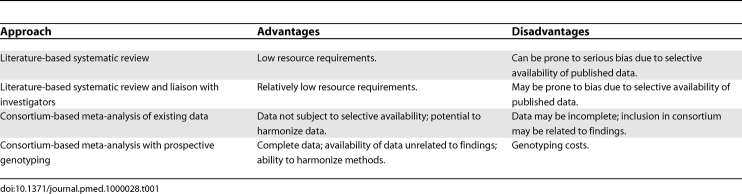
Data Sources for Meta-Analysis of Genetic Association Studies

## Preparation of a Protocol

Like other research studies, systematic reviews should ideally be carefully planned with a detailed protocol prepared in advance. Producing a protocol with criteria predefined for study selection will minimize any selection bias based on study results. Such a protocol should therefore clearly formulate the review question, explain the rationale for conducting such a review, define a priori eligibility criteria for study inclusion, describe the methods for conducting a comprehensive search for studies, indicate the methods for assessing study validity and relevance, and state whether a meta-analysis is planned and describe the methodology to be used for conducting such an analysis.

## Objectives and Eligibility Criteria

The objectives of a systematic review of genetic association studies will typically be (i) to identify all epidemiological investigations of the associations of interest; (ii) to assess the validity of the evidence; (iii) to determine whether an association exists; (iv) to assess whether associations are consistent across studies in magnitude and direction; and (v) to quantify the likely magnitude of an association if it exists. Associations of interest have in the past usually been identified through biological plausibility, although candidate associations are now frequently identified from genome-wide association studies.

The applicability of a systematic review depends on the selection of appropriate studies to be included in it. Eligibility criteria for the review, defined a priori, should allow relevant studies investigating the gene(s), allelic variant(s), disease(s), clinical subtypes, and any other outcomes of interest (such as molecular biomarkers) to be easily identifiable and included in the review. Appropriate study designs and study populations should also be considered in order to allow the reliable assessment of associations and interactions.

## Searching for Eligible Studies

A key characteristic of a systematic review is a comprehensive search. Limiting a review to studies identified only from MEDLINE (or PubMed) is usually insufficient, for two reasons. First, any review restricted to published literature is prone to publication bias, whereby only a subset comprising the most “publishable” findings are available from the totality of evidence on a particular genetic association. Thus, sources beyond the published literature should be examined. Second, even within the published literature, reporting bias may be present whereby studies with different conclusions are published in different types of journals. MEDLINE is just one of several major sources of bibliographic information. These biases may, to some extent, be reduced by searching comprehensively for studies available through other sources. Other bibliographic databases likely to be useful for genetic association studies include EMBASE and the Science Citation Index. Overlap between these various databases is far from complete; for example, of approximately 4,800 journals indexed in EMBASE, 1,700 are not indexed in MEDLINE (see Chapter 6 in [9]). The implications of this incomplete overlap for genetic association studies are not clear. Furthermore, bibliographic searches may still not retrieve all articles that are in the indexed journals [12].

Further sources that might be searched include reference lists of study reports and review articles, Web sites, theses, books, reports, and online databases (including data repositories for genome-wide and other association studies [13]). Sources other than peer-reviewed journals are often referred to as “grey literature.”

**Figure 2:**
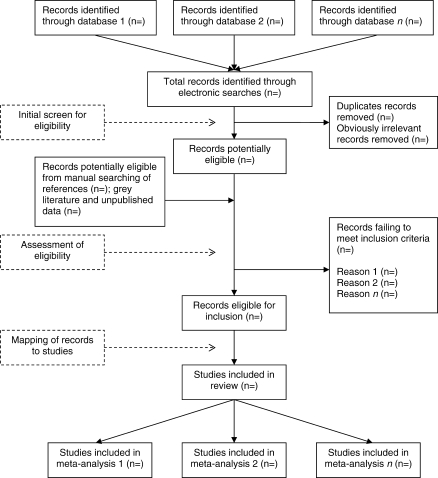
A Typical Process for Searching and Assessing Eligibility of Studies

Once the searches have been undertaken, the records identified need to be assessed against the eligibility criteria for the review. A typical example of the process from search to selection is illustrated in Figure 2.

## Minimizing Human Error

All reasonable attempts should be made to prevent the introduction of errors and personal biases. An important attribute of systematic reviews is that criteria for study inclusion are clearly prespecified. This is perhaps the most notable difference between a narrative review and a systematic review. Independent duplication of steps in the review process, such as selection of studies, extraction of data, and critical assessment of methods used in the individual studies, can further reduce biases and minimize errors. Accidental omission of data, or accidental duplication of a study in a meta-analysis, may lead to spurious false-negative or false-positive findings.

## Assessing Validity of Each Study

The validity of a meta-analysis depends on the validity of the studies included within it, so it is important that each component study is appraised before being included. Because effect magnitudes in genetic association studies are generally small (i.e., odds ratio < 1.2), even small biases may be important. The most important sources of bias in genetic association studies are less well understood than those in other study designs such as randomized trials. Extensive discussions of potential biases are available, with the principal candidates being case definition, population stratification (confounding due to sub-populations in the sample that differ both in genotype prevalence and disease risk), and methods in the collection, handling, and processing of DNA and the determination of genotypes (including blinding to case-control status) [3,14]. Little empirical evidence associating study results with study characteristics for genetic association studies exists as yet, however. This evidence will perhaps be most reliably derived from meta-epidemiological studies, in which results of studies with different characteristics are compared within meta-analyses, and findings are synthesized across meta-analyses to enhance power [15,16].

The appraisal of potential biases is difficult in practice, not only because of the uncertainty over which study characteristics are important, but also because of incomplete or variable reporting of the methods used in the studies themselves [17]. Initiatives such as the STREGA (STrengthening the REporting of Genetic Association Studies) statement, which offers guidelines for reporting of individual genetic association studies, may improve the situation in the future.

## Assessing Bias in the Review as a Whole

In addition to potential biases in the individual studies, attention should be paid to bias in the collection of studies as a whole. Two particular considerations are reporting biases and bias in the selection of genetic variants to study.

Reporting biases include both publication bias, which typically refers to suppression of evidence concerning an entire study on the basis of its findings, and selective reporting, whereby only the most exciting findings are reported. Selective reporting of only the most promising variants is a natural consequence of an attempt to summarize an association study of many genetic markers within the constraints of a traditional paper journal article. To overcome these constraints, association studies are increasingly exploiting Web-based publication for complete findings, although in practice there are often obstacles to prevent this occurring.

The possibility of reporting biases can only be discarded if it is known that all eligible data from all eligible studies are available (or if a truly unbiased subset of these data is available). It is difficult to imagine situations in which reviewers are confident that all existing studies are either known or reported without bias. The most reliable way to overcome these biases is therefore in the prospective generation of data, for example by using a consortium-based approach.

It is also helpful to keep in mind that the very act of investigating a gene-disease association may be dependent on earlier results. Even in a world of flawless studies that are completely reported, it is likely that the most exciting findings are those that are targets for replication. Initial exciting findings may well be chance effects, and so situations in which a meta-analysis is contemplated (those in which multiple studies exist) comprise a biased subset of possibly important associations. There is good evidence of the exaggerated effect that is often seen in the earliest report of an association compared with subsequent attempts to replicate the finding [18].

## Meta-Analysis

**Figure 3:**
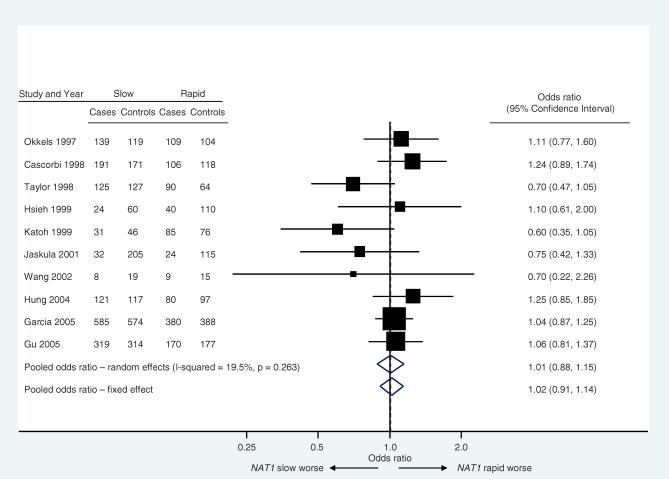
Example of a Forest Plot The plot shows the relation between *N*-acetyltransferase (*NAT1*) acetylation status (slow or rapid) and bladder cancer risk (data from Sanderson et al. [20]). The classification of acetylation phenotype is made either directly, by measuring the metabolism of a probe drug, or indirectly, by measuring the genotype associated with that phenotype.

Meta-analysis is the statistical synthesis of results from multiple studies [19]: an example is provided in Figure 3 [20]. When implemented and interpreted appropriately, and applied to unbiased and correctly analyzed studies, it provides a powerful tool to understand both similarities and differences in results from multiple studies. By exploiting the totality of evidence, meta-analyses offer enhanced power to detect associations and increased precision in the estimation of their magnitude. Meta-analyses are encouraged in systematic reviews. However, attention should always be paid to the possibility of reporting biases in reviews based on published literature. Furthermore, it is important that each entry in a meta-analysis represents an independent sample of data. Thus for example, multiple reports of the same study need to be merged to obtain a single “best” answer for that study prior to inclusion in the meta-analysis.

Most meta-analyses are undertaken by calculating weighted averages of the estimates from multiple studies. A metric is chosen for the analysis that ensures comparable quantities with reasonable statistical properties. For example, log odds ratios are typically combined for case-control studies (and then presented as odds ratios). If quantitative traits are measured using different methods across studies, they may be standardized before pooling, usually by expressing the results in terms of standard deviations.

Consistency of results across studies should always be evaluated, and can be achieved using a statistical test of homogeneity or by quantifying the between-study variance (or quantities derived from these such as *I* ^2^, which describes the proportion variation in point estimates that is due to heterogeneity rather than within-study error [21]). Potential reasons for variation in findings across studies should also be investigated. To minimize unreliable consequences from multiple testing, potential sources of variation in study results should be limited in number and prespecified whenever possible. Consideration should be given to using methods to temper the statistical significance of findings based on a small number of studies [22].

Sensitivity analyses are to be encouraged, to ensure that findings are robust. For example, undue influence of the initial study, of smaller studies, or of studies with potential biases may be responsible for inappropriate conclusions. Furthermore, the choice of statistical model (or method) or the inclusion of studies with uncertain eligibility for the systematic review may affect the results, and robustness to these should also be evaluated.

Meta-analysis methods of gene-disease association studies closely follow well-developed methodology for randomized trials [4,6,23]. Due to so-called “Mendelian randomization” in the transmission of genetic material from parents to children, confounding is generally thought to be of minimal concern (although not ignorable, since it may arise through population stratification). Thus, unadjusted analyses are common in meta-analyses, even if matched studies have adjusted for matching factors such as age and sex.

The era of genome-wide association studies has implications for the meta-analysis of genetic associations. Although, in principle, summary data for the variant(s) of interest in the review should be sought from available genome-wide association studies in the same way as they are sought from candidate gene studies, in practice many genome-wide association studies are designed primarily for detecting associations rather than quantifying them, and the potential for bias must be assessed. Furthermore, the variant(s) of interest might not have been included in a genome-wide scan. While it is possible that some unknown genotypes could be imputed, proposed methods typically require detailed data [24], and it is unclear whether a more naive approach, based on minimal summary data and linkage disequilibrium information, would be appropriate.

Other special considerations in the meta-analysis of gene-disease associations are discussed in detail elsewhere and include the choice of inheritance model [25,26], the treatment of Hardy-Weinberg equilibrium [27,28], and the combination of associations for markers known to be in linkage disequilibrium [29]. Developments in the last area are now allowing complex syntheses of data across whole-genome-wide association studies [30].

## Presenting and Interpreting Results

A systematic summary of all available evidence should allow the strengths and gaps in the evidence base to be identified and allow recommendations to be made in order to stimulate research to address such gaps. The magnitude of the association between the allelic variants and the clinical outcomes studied in terms of relative and attributable risks in different populations should be summarized in a systematic and concise way. Comments should also be provided on the quality and methodology of studies. Tables should summarize information on each gene-disease association study (possibly online as Web-based supplements, depending on size and journal formats). Table 2 lists some of the characteristics most likely to be relevant. If a meta-analysis is conducted, these should usually be presented as forest plots as in Figure 3. Statistics related to heterogeneity (e.g., between-study variance or *I* ^2^ and confidence intervals) and investigations of bias should be presented. Multiple subgroup and sensitivity analyses are conveniently presented as “overview” forest plots without including the individual studies.

**Table 2:**
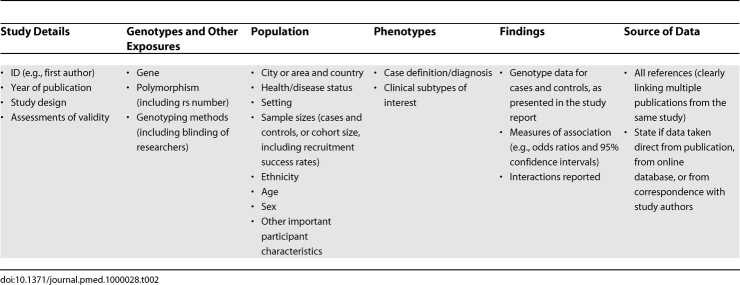
Characteristics Likely To Be of Interest for Each Study Included in a Review

A discussion of the results should also address issues such as the overall quantity and quality of the evidence base, the consideration of possible publication and selective reporting biases, the likelihood that associations are causal, and any potential public health applications. For example, results from small or few studies should be interpreted with great caution, as it is common to see dissipation of early claimed effects [31], and early studies have no predictive power for the subsequent picture of the evidence [32]. Interim guidance is available to assess the strength of evidence that an observed association is genuine [33]. These criteria assess the three domains of precision (i.e., through sample size), consistency of results across studies (i.e., through meta-analytic measures of heterogeneity), and protection from bias both within and across studies in a meta-analysis.

Additional detailed guidance for the general reporting of systematic reviews and meta-analyses are available from PRISMA (Preferred Reporting Items for Systematic Reviews; formerly known as QUOROM) [34]. Reporting guidance for meta-analyses of observational studies are also available from MOOSE (Meta-analysis Of Observational Studies in Epidemiology) [35].

## Concluding Remarks

The strengths and limitations of systematic reviews are well established for clinical trials, largely through the efforts of The Cochrane Collaboration [36]. They are increasingly being applied to observational studies, and currently there are as many meta-analyses of observational data conducted as there are of clinical trials. The citation impact of both types of meta-analyses is equally high, the highest among all study designs in the health sciences [37]. The principal value of a well-conducted systematic review of genetic association studies is in establishing reliably the presence and magnitude of individual gene-disease associations. By complementing both consortium-based pooled analyses and larger-scale attempts to collate genetic association evidence across whole fields, they play an important role alongside other research designs in the integration of evidence on genetic association [5]. At their most ambitious, systematic reviews and meta-analyses can collate evidence across all studied genetic variants for a phenotype, with notable examples provided by three databases of genetic association evidence for Alzheimer disease, Parkinson disease, and schizophrenia that include ad hoc meta-analyses [38–40].

Within the Summary Points we summarize our key suggestions for methodology of systematic reviews of genetic association studies. These reflect our accumulated experience to date and are not intended to lay down standards for universal application. This is an area of rapid development. In a decade's time, appropriate methods for the collation and integration of evidence on gene-disease association may be substantially different.

## Abbreviations

HuGENet: Human Genome Epidemiology Network
